# Cooperative Data Collection Mechanism Using Multiple Mobile Sinks in Wireless Sensor Networks

**DOI:** 10.3390/s18082627

**Published:** 2018-08-10

**Authors:** Weimin Wen, Chih-Yung Chang, Shenghui Zhao, Cuijuan Shang

**Affiliations:** 1 School of Computer and Information Engineering, Chuzhou University, Chuzhou 239000, China; wenweim@chzu.edu.cn (W.W.); zsh@chzu.edu.cn (S.Z.); shangcuijuan@chzu.edu.cn (C.S.); 2 Department of Computer Science and Information Engineering, Tamkang University, New Taipei City 25137, Taiwan

**Keywords:** collection point, network lifetime, rendezvous time, energy consumption

## Abstract

Data collection problems have received much attention in recent years. Many data collection algorithms that constructed a path and adopted one or more mobile sinks to collect data along the paths have been proposed in wireless sensor networks (WSNs). However, the efficiency of the established paths still can be improved. This paper proposes a cooperative data collection algorithm (CDCA), which aims to prolong the network lifetime of the given WSNs. The CDCA initially partitions the *n* sensor nodes into *k* groups and assigns each mobile sink acting as the local mobile sink to collect data generated by the sensors of each group. Then the CDCA selects an appropriate set of data collection points in each group and establishes a separate path passing through all the data collection points in each group. Finally, a global path is constructed and the rendezvous time points and the speed of each mobile sink are arranged for collecting data from *k* local mobile sinks to the global mobile sink. Performance evaluations reveal that the proposed CDCA outperforms the related works in terms of rendezvous time, network lifetime, fairness index as well as efficiency index.

## 1. Introduction

Wireless sensor networks (WSNs) have been used in many applications, including healthcare, trajectory tracking, environmental monitoring, smart home, military surveillance, coverage, rechargeable and data collection [1,2,3,4,5,6,7,8,9]. The data collection issue has received much attention in recent years. In literature, many studies develop data collection approaches, aiming to cope with the energy unbalanced issue in a given region. An advanced routing transfer-low-energy adaptive clustering hierarchy approach (ART-LEACH) has been proposed in [10]. It assumed that the sensor nodes and sink node are static. These studies have minimized energy consumption and extended network life. But the closer the sensor nodes are to the sink, the greater the data-forwarding workloads and power consumption of the node, leading to an energy imbalance problem.

To deal with energy imbalances issue, numerous studies have adopted mobile sink to collect data from sensor nodes. These studies are mainly classified into two categories: single mobile sink and multiple mobile sinks. In the first class, some studies [11,12] adopted one mobile sink to patrol all sensor nodes and collect their readings. Since there is no data forwarding, the power consumption of sensor nodes are minimized. However, the length of the constructed path is too not efficient, raising the problems of buffer overflow of sensor nodes.

To resolve the path inefficient problem, some other studies [13,14,15] fall in the partial data-forwarding class, which uses a mobile sink to pass through only some nodes. All the visited sensors were called collection points (CPs). Most of these mechanisms partitioned the sensor nodes into several groups and then established a subtree for each group, where the root of each subtree was a CP. Along the topology of the subtree, each sensor node transmitted its data to the root. Under the given constraint of the total path length, the mobile sink built a route passed through the roots of all subtrees. Then the mobile sink treated these roots as data collection points and collected data from them. However, the selected CPs might not be appreciated, increasing the time required for data collection and reduces the life time of the network in some large-scale applications.

To avoid the occurrence of this situation, some other studies [16,17,18,19,20] fall in the class of multiple mobile sinks. All sensor nodes were geographically divided into several clusters. Based on the remaining energy of each sensor node, a cluster header will be selected in each cluster. Each mobile sink was responsible for collecting data for each cluster. Each mobile sink will establish a route which passed through all nodes in a cluster. The mobile sink then traversed the route and collected data from these nodes in the cluster. However, most of them did not take into account the length cost between any two consecutive cluster headers, the velocity control of mobile sinks and the cooperation between the mobile sinks. As a result, the latency of data collection was long and the established route still can be improved.

Given *k* + 1 mobile sinks and a set of *n* sensor nodes, this study presents a cooperative data collection algorithm, called CDCA, which initially partitions the *n* sensor nodes into *k* subsets $T=T_{1},T_{2},\ldots ,T_{k}$ and assigns one local mobile sink $m_{i}$ to collect the data generated by sensors in $T_{i}$. Then the proposed CDCA aims to select $k_{i}$ CPs from the sensors in $T_{i}$ and establishes a route $\pi_{i}$ which passes through the $k_{i}\;$ CPs for data collection. To further collect all data from the *k* mobile sinks, the (*k* + 1)-th mobile sink, called global mobile sink, will construct a path where the *k* local mobile sinks can rendezvous with the global sink on the path and transmit the collected data to the global sink. This can improve the energy consumption of sensor nodes and can maximize the network lifetime of the WSNs. The contributions of this paper are itemized as follows.

- **Achieving the purpose of cooperative data collection.** The proposed CDCA partitions the data collection task into *k* subtasks through benefit calculations. The mobile sinks can cooperatively collect data along the constructed paths.
- **Prolonging network lifetime.** The proposed CDCA considers the forwarding workload of each sensor, selects the *k* sensors with the largest forwarding workloads and then partitions a tree with *n* sensors into *k* subtrees. This can minimize the energy consumption of sensors with maximal energy consumption and hence prolong the network lifetime.
- **Balancing the workloads of sensors for packet forwarding in each subset.** The proposed mechanism considers the length cost between any two consecutive CPs. Therefore, the established route allows the mobile sink to visit more collection points. Compared with weighted rendezvous planning (WRP) [15], the proposed CDCA distributes the workloads of data forwarding to more collection points, prolonging the network lifetime.
- **Maintaining a stable cycle for the rendezvous opportunities between each local mobile sink and global sink.** The proposed mechanism adjusts the velocities of the mobile sinks to ensure that each local mobile sink and the global sink can have stable rendezvous time periodically.

The remainder of this work is organized as follows. Section 2 reviews the existing works related to this study. Section 3 illustrates assumptions, network environment and the problem formulation of the proposed approach. Section 4 presents the proposed mechanism. The experimental results of the proposed mechanism are proposed in Section 5. Finally, Section 6 offers a conclusion and future works.

## 2. Related Work

In literature, a number of studies that adopted mobile sink for collecting data are mainly classified into two categories, including single mobile sink collecting data [11,12,13,14,15] and multiple mobile sinks collecting data [16,17,18,19,20]. The following briefly reviews these related works.

Studies [11,12,13] fall in the first class mainly used single mobile sink to collect data from the sensor nodes. Study [11] proposed a heuristic tour-planning schedule for single-hop data collection. This study aims to decrease path length but visit all sensor nodes. However, the algorithm may raise the problem of long delay in large-scale sensor network. In [12], a mobile sink collects data from all sensor nodes in a given wireless sensor network. The main objective of this algorithm is to minimize the latency of data collection by designing the shortest path which passes through all sensor nodes. Somasundara et al. [13] proposed an algorithm to build path of the mobile sink for collecting all the data before the buffer of each sensor node overflows. However, with an increasing number of sensor nodes, it will still become impractical due to the high computational complexity.

The previously mentioned studies visiting all sensors become impractical when the number of sensor nodes grows. In order to cope with the data collection issue, some other works [14,15] fall in the partial data-forwarding category where the mobile sink only visits some sensor nodes. Those nodes that are not visited by mobile sink should forward their data to the closest visited sensors. Yi-Hsuan et al. [14] proposed a heuristic algorithm which consists of two steps. The first step is finding the root of a data aggregation tree such that the hop distance from any node to the root is minimized. Then the second step selects sensors as collection points to be visited by the mobile sink for data collection. However, the heuristic path construction algorithm did not consider that the distance between current CP and the next CP. This might construct a long path for the mobile sink.

Hamidreza et al. [15] proposed a weighted rendezvous planning (WRP) where each sensor node is assigned a weight corresponding to its hop distance from the tour and the number of data packets that it forwards to the closest CPs. Then a set of CPs is determined for constructing a near-optimal traveling tour that minimizes the energy consumption of sensor nodes. However, it did not consider the path cost between two consecutive CPs. This reduces the number of sensors visited by the mobile sink and hence causes the problems of shorter network lifetime or fewer collected data.

The abovementioned studies used only single mobile sink to visit each sensor node and collect its data. When the size of network area or the number of sensors grows, the path length of the mobile sink must be increased accordingly. The mobile sink will spend much more time for each round and hence cause high latency for data collection. Therefore, a single mobile sink may not be sufficient for certain applications that require low latency in a large-scale WSN. There have also been several studies [16,17,18,19,20] that use multiple mobile sinks for data gathering in WSNs to reduce latency. The following reviews these studies.

Zhao et al. [16] straight lines and receive data sent from sensors via multihop transmission. In case the sensors are deployed uniformly and the density is high, the algorithm has good performance. However, if some sensors in the network is blocked by obstacles or holes, the performance will be inefficient because that the mobile sinks only move along straight lines. To cope with this problem, Aslanyan et al. [17] adopted multiple mobile sinks that randomly move in the monitoring region for data collection. In addition, the mobile sinks delivered the received data to the other mobile sink when they fall in the communication range. Since the route is randomly determined, the latency is difficult to be estimated and is unstable.

Stefano et al. [18] proposed an energy efficient clustering with delay reduction in data gathering scheme that focuses on energy efficient routing of data from sensor nodes to base station using multiple mobile sinks. This increased the network lifetime, throughput, and delivery rate. However, the proposed mechanism did not consider the issue of speed control for the mobile sinks and hence the cooperation between mobile sinks are not mentioned. Edison et al. [19] presented a bio-inspired networking strategy to allow the cooperation of static sensors on the ground and multiple mobile sensors in the air, applied to the scenario of large areas surveillance. The strategy can provide efficient communication among the sensor nodes, reduce the number of messages exchanged in the network. However, the proposed mechanism did not consider the issue of speed control for the mobile sinks and hence the cooperation between mobile sinks are not mentioned.

Duc Van et al. [20] proposed a data collection scheme, called HiCoDG, which adopted multiple mobile sinks to collect and relay data in a cooperative manner. The proposed HiCoDG algorithm aims to find the optimal paths for mobile sinks and global mobile sink, for minimizing the traveling distances of mobile sinks. However, the proposed mechanism did not consider the balance of the number of nodes in the cluster and the distance between the current cluster-head and the next cluster-head.

Most of the studies mentioned above emphasize the improvement of the data fresh problem. But most of them did not take into account the velocity control for mobile sinks and the cooperation between the mobile sinks. As a result, the latency of data collection might be increased and the constructed path is not efficient enough. This paper proposes a cooperative data collection algorithm, called CDCA. Compared with the existing studies, the proposed CDCA exhibits several contributions, including achieving the purpose of cooperative data collection, prolonging network lifetime, balancing the workloads of sensors for packet forwarding and maintaining a stable cycle for the rendezvous opportunities between each mobile sink and global sink. The following summarizes the comparisons of related works and the proposed CDCA, in terms of several important parameters which impact the performance of the network lifetime of the given sensor network and cooperation between the mobile sinks.

Table 1 compares each related work with this paper in terms of number of mobile sinks, path construction, data collection latency and mobile sink cooperative. It is shown that the proposed mechanism exhibits all good characteristics, as compared with the other six mechanisms.

## 3. Network Environment and Problem Formulation

This section introduces the network environment and assumptions of the modeled WSN. Then the problem formulation is presented.

### 3.1. Network Environment

Given a monitoring region Ɽ, this paper considers a mobile wireless sensor network that consists of a set of *n* sensor nodes $S=\left\{s_{1},s_{2},s_{3},\cdots ,s_{n}\right\}$ randomly deployed in Ɽ. There are *k +* 1 mobile sinks $M=\left\{m_{0},m_{1},\cdots ,m_{k}\right\}$ aiming at collecting data from all sensor nodes. The *k* mobile sinks, including$\;m_{1},\ldots ,\;m_{k}$, aim to collect data from all sensors in their own local area while the$\;\;m_{0}$ treated as the global mobile sink aims to collect data from the *k* mobile sinks. The global mobile sink $m_{0}$ is initially arranged at the location of static sink. The function of static sink is to upload the data to the Internet for further processing. Assume that the communication ranges of all mobile sinks and sensor nodes are identical. Each mobile sink has rich power or is supported with an energy harvesting system. In addition, the paper assumes that all mobile sinks are aware of the locations of all sensor nodes and their own location information. Among the *k* mobile sinks, there is a leader, denoted by $m_{leader}$, which is in charge of executing the proposed data collection mechanism and broadcasting the results to all mobile sinks.

All sensor nodes periodically generate one data packet in every time period *t* and the packet must be delivered to the sink. This paper aims to develop a data collection mechanism which partitions the sensors into *k* disjoint sets $T=T_{1},T_{2},\ldots ,T_{k}\;$ and assigns *i*-th set $T_{i}$ to mobile sink $\;m_{i}$. Since the mobile sink $m_{i}$ aims to collect data from all sensor nodes in $T_{i}$, it establishes a tour passing through all sensors in $T_{i}$. However, for some large-scale applications, it is time consuming for data collection, which might lead to the problem of buffer overflow. To prevent from this situation, the leader should select some sensors and only visit them in $T_{i}.$ Let $P_{i}=\left\{p_{i,1},p_{i,2},p_{i,3},\cdots ,p_{i,k_{i}}\right\}$ be the set of $k_{i}$ selected CPs in $T_{i}$. In each round, the mobile sink $m_{i}$ will visit each $p_{i,j}$ and collect data from each $p_{i,j}$ and then return to the root of $T_{i}$. Let ${\hat{\pi }}_{i}$ represent the path passing through all CPs $p_{i,j}$ in $T_{i}$ and the path length of $\pi_{i}$ is $\left|\pi_{i}\right|$. In the next subsection, the problem formulation of this paper will be presented.

**Figure 1:**
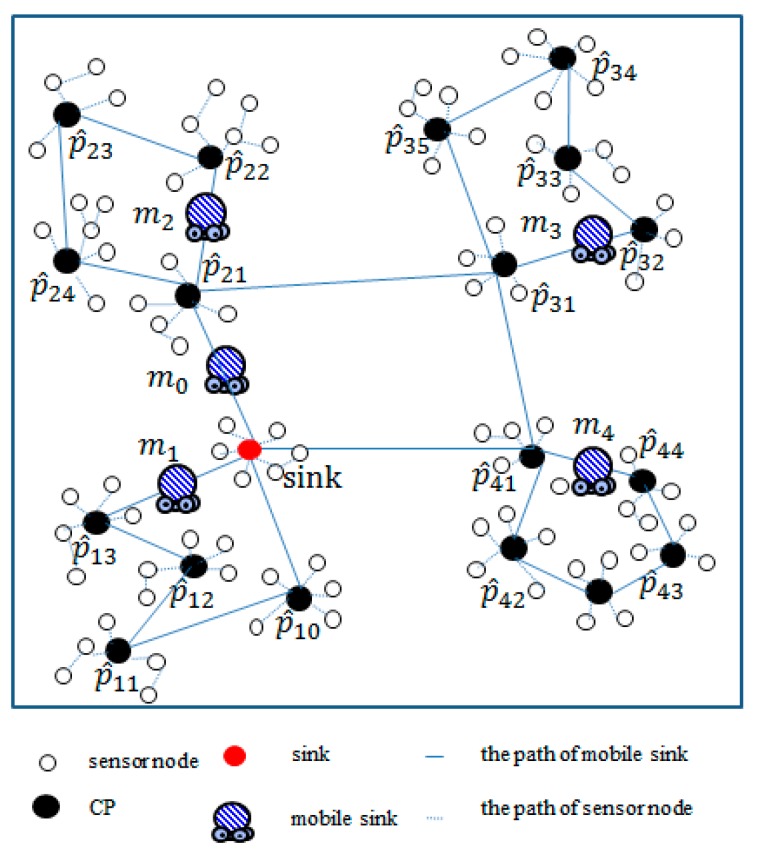
An example of the considered network environment.

Figure 1 illustrates an example of several CPs and five mobile sink in a rectangular region. In Figure 1, the red circles represent the static sink whereas the black circles denote the selected CPs. The sensor nodes have been partitioned into five groups, and each group organizes a path by $m_{i}$. In each path, every mobile sink $m_{i}\;$ moves along the established path, which passes through all CPs in $T_{i}$ to collect data. The global mobile sink $m_{0}$ will leave from the static sink, move along the constructed path and collect all data stored in root $T_{i}$. Then the global mobile sink $m_{0}$ goes back to the static sink.

### 3.2. Problem Formulation

Energy conservation is the most important parameter that determines the lifetime of a wireless sensor network. In general, the lifetime of a given WSN is defined by the time length starting from the time point that the network operates to the time point that the first sensor node runs out its energy. This paper aims to develop a data collection mechanism which initially partitions the *n* sensor nodes into *k* subsets $T=T_{1},T_{2},\ldots ,T_{k}$ and assigns one mobile sink $m_{i}$ to collect data generated by sensors in $T_{i}$. Then the data collection mechanism aims to select $k_{i}$ CPs from the locations of sensors in $T_{i}$ and establish a route $\pi_{i}$ which passes through the $k_{i}$ CPs for data collection. Since the CPs are bottlenecks of network lifetime, saving their energy consumption can prolong the network lifetime. 

In the following, the energy consumption model considered in this paper is [21]. The energy consumption of wireless sensor nodes mainly happen on the operations including data sending and receiving. Let sender $s_{i}$ and receiver $s_{j}$ be a communication pair and $s_{i}\;$sends *k* bits to $s_{j}$. Let $d_{ij}$ be the distance between $s_{i}$ and $s_{j}$. The energy consumption for each sensor node receiving δ bits can be measured by Equation (1).

(1) $$\;\;\;\;\;E_{R}=\delta \beta \;\;\;$$

where β is the parameter indicating energy consumption for transmitting one bit. The energy consumption for sender $s_{i}$ to transmit a packet to its parent $s_{j}\;$ is expressed as Equation (2).

(2) $$\;E_{T}=\delta \varepsilon_{1}\;+\;\delta d_{i,j}^{\gamma }\varepsilon_{2}\;$$

where $\varepsilon_{1}$ is a parameter denoting the energy consumption for transmitting one bit, $\varepsilon_{2}$ is the energy consumption factor of the amplifier circuit, $d_{i,j}^{\gamma }$ is the distance between sensors $s_{i}$ and $s_{j},$ γ  is the path-loss exponent, which usually ranges between 2 and 4, depending on the environment. 

The lifetime of sensors in $T_{i}$ highly depends on the value of $k_{i}$. A large value of $k_{i\;}$ indicates that the mobile sink $m_{i}$ can visit more CPs. This implies that all sensors in $T_{i}$ can be further partitioned into $k_{i}$ subsets. Each subset will be associated with a CP and all sensors in this subset should transmit their data to this CP. Consider each subset and the corresponding CP. Since the distance between each sensor and the CP might be larger than the communication range, a tree rooted by CP will be constructed for relaying the data from each sensor to CP along with the tree topology. Let subtree $T_{ij}$ is rooted by CP $p_{ij}$ and has $u_{ij}+\;1$ nodes. Sensor nodes of each subtree $T_{ij}$ should upload their data to the root $p_{ij}$. These roots will store the data and then transmit the data to mobile sink when the mobile sink visits them. A route that visits all CPs should be constructed for each mobile sink $m_{i}$ to collect data from all CPs in $T_{i}$. The energy consumption of sensor nodes mainly happen on the operations including data transmitting and receiving. Let $E_{ij}$ denote the energy consumption of $p_{ij}$ for receiving one packet from its children. Equation (3) evaluates the value of $E_{ij}$ which equals to the sum of energy consumption for receiving $u_{ij}$ packets and transmitting $u_{ij}+\;1$ packets in tree $T_{i}$.

(3) $$\;\;E_{ij}=u_{ij}\;\times \;E_{R}\;+\;\left(u_{ij}\;+\;1\right)\;\times \;E_{T}\;$$

The network lifetime is determined by the lifetime of the sensor with the least energy. Since each CP consumes the highest energy in its tree, this paper aims to reduce the energy consumption of the CP which consumes maximal energy as compared with the other CPs. Let $s_{ij}^{max}$ denote the bottlenecked CP in $T_{i}$. The value of $s_{ij}^{max}$ can be derived by applying Equation (4)

(4)  sijmax=arg(ETi∈T,pij∈TiMaxij) 

Expression (5) reflects the goal of this paper.

Objective:

(5)  s1≤i≤kMin  Max   ijmax 

When achieving the objective (5), the following constraints (6)–(9) must be satisfied. Let |L| denote the length of path  L. Let $\;L_{min}$ denote the shortest Hamilton path passing through every CP in Tree $T_{i}$ and $L_{max}$ denote the maximal length of a valid path. Let $\;d_{i}\;$denote the rendezvous point of global mobile sink $m_{0}$ and local mobile sink $m_{i}$. Let path $\pi_{0}$ denote the path that passes all rendezvous points. Constraint (6) illustrates that the length of each path $\pi_{i}$ should be smaller than or equal to the maximal length that mobile sinks can move and larger than the minimal length.

(1) Distance Constraint:

(6) $$\;\left|\;L_{min}\right|\;<\;\left|\pi_{i}\right|\;\leq \;|L_{max}|,\;0\;\leq \;i\;\leq \;k\;$$

Each mobile sink $m_{i}$ should collect data from all CPs in $T_{i}$ and then arrives at the rendezvous point $d_{i}$ to forward its data to the global sink $m_{0}$. Let $m_{0}$ and $m_{i}$ arrived at rendezvous point $\;d_{i}$ at time points $t_{i}^{1}$ and $t_{i}^{2}$, respectively. To guarantee that the collected data are fresh, the *rendezvous delay constraint*, as shown in Constraint (7), should be satisfied.

(2) Rendezvous Delay Constraint:

(7) $$\;\;t_{i}^{1}\;-\;t_{i}^{2}\;\leq \eta \;\;$$

where *η* is an acceptable time delay at each rendezvous point.

Let one round, denoted by notation $\varsigma_{i}$, represent the time period required for collecting one data packet from each sensor in $T_{i}$. Let Ω denote the lifetime of a given WSN. The number of rounds that mobile sinks can visit $T_{i}$ is $\;\Omega /\varsigma_{i}$. Since the energy consumption of $p_{ij}$ is $E_{ij}$ in each round. The total energy consumption of CP $p_{ij}$ is $E_{ij}\;\times \;\left(\Omega /\varsigma_{i}\right)$. Constraint (8) checks if the total energy consumption is less than or equal to the battery capacity *B*.

**(3) *Battery Constraint:***

(8) $$\;E_{ij}\;\times \;\left(\frac{\Omega }{\varsigma_{i}}\right)\leq \;B\;\;\;\;\;1\;\leq \;i\;\leq \;k,\;1\;\leq \;j\;\leq \;k_{i}\;$$

where  B denotes the battery capacity.

Let $S_{ij}$ denote the set of sensors rooted by CP $p_{ij}$ in subtree $T_{ij}$. Let $f_{ij}^{out}$ denote the total number of packets sent by CP $p_{ij}$ in each round and let $f_{i}^{in}\;$ denote the number of packets created by each $s_{i}$ in set $S_{ij}$ in each round. To guarantee that all packets of $S_{ij}$ can be received by the CP $p_{ij}$, and then $p_{ij}$ forwards the received packets and its own packet to the mobile sink, the following Flow Constraint (9) should be satisfied.

(4) Flow Constraint:

(9) $$\;\;f_{ij}^{out}=(\underset{\forall s_{i}\in S_{ij}}{\sum }f_{i}^{in})\;+\;1\;$$

## 4. The Proposed CDCA Algorithm

This section proposes the data collection mechanism which aims to partition the whole wireless sensor network into *k* disjoint sets and assign each set to a mobile sink $m_{i}$ to collect the data generated in this set. For each set of sensors, the proposed mechanism will further determine the set of CPs and establish a path visiting all CPs while the lifetime of the sensors in this set can be maximized, under the constraints (6)–(9). The proposed data collection algorithm is composed of three major phases: Network Partition Phase, CP Selection and Path Construction Phase as well as Speed Control Phase. In the Network Partition Phase, the algorithm partitions the n sensor nodes into k disjoint subsets. Then the CP Selection and Path Construction Phase aim to select $k_{i}$ CPs from the locations of sensors in $T_{i}$ and establish a path $\pi_{i}$ which visits the $k_{i}$ CPs for data collection. In the *Speed Control Phase*, the algorithm coordinates to control the speed of mobile sinks $m_{0}\;$and $m_{i}.$ As a result, each mobile sink $m_{i}$ can collect data generated by sensors in $T_{i}$ while the mobile sink $m_{0}$ further collect data from each mobile sink $m_{i}$. The following gives the details of the three phases.

### 4.1. Network Partition Phase

Given a _mi_nimum spanning tree (MST) T, the root of tree *T* can be considered as the sink and there is a mobile sink $m_{0}$ located at the root. The Network Partition Phase aims to partitions T into *k* disjoint subsets $T=T_{1},T_{2},\ldots ,T_{k}$ such that the sizes of them are similar. Each mobile sink $m_{i}\;$ will be allocated to $T_{i}$ for data collection. Let S denote the set of all sensor nodes. Let Ñ and $\hat{S}=S\backslash Ñ$ denote the sets of selected and not selected roots for *k* subtrees, respectively. Let $s^{largest}$ denote the sensor with largest cost in $\hat{S}$. The basic concept of this phase is to find the sensor $s^{largest}$ in $\hat{S}$ in each run to play the role of tree root until *k* roots are selected. In each run, the selected $s^{largest}$ will join set Ñ. After that, the minimum spanning tree will be restructured. The following gives the general descriptions for the sink to split up the MST T.

Let D(i,T) represent the number of hops from sensor $s_{i}$ to the root of its tree. Let NS(i,T) denote the number of sensors rooted by $\;s_{i}$. Assume that each sensor creates one packet in each round. Let NP(i) represent the number of packets received by sensor node$\;s_{i\;}$, including its own reading. Equation (10) presents the relation of NP(i) and NS(i,T).

(10)  NP(i)=NS(i,T) + 1 

Let Hops(i,sink) denote the number of hops from $s_{i}$ to the sink contains sensor node $\;s_{i}$. If sensor $s_{i}$ is selected as the root of some subtree $T_{i}$, the number of packets saved by selecting $s_{i}$ as the *CP* is evaluated as shown in Equation (11).

(11)  D(i,T)=Hops(i,sink) × NP(i) 

Let $W_{i}$ denote the cost obtained by selecting $\;s_{i}$ as the tree root. The value of $W_{i}\;$ is measured by the number of packets saved for transmission from some $\;s_{j}\in P$ to$\;s_{i}$, as shown in Equation. (12).

(12) $$\;W_{i}=D\left(i,T\right)\;\times \;NP\left(i\right)\;$$

The sink calculates the $W_{i}\;$ of each $\;s_{i}\in \hat{S}$ and selects the sensor $s^{largest}$ with the largest cost. Expression (13) reflects the condition of sensor, which can be selected as the tree root.

(13) $$\;s^{largest}=arg\underset{\;s_{i}\in \hat{S}}{\max}W_{i}\;$$

After that, the sensor $s^{largest}$ is added into Ñ and is removed from $\hat{S}$. The aforementioned operations will be repeated executed round by round until *k* roots are selected in Ñ. As a result, the final selected largest cost set $Ñ=\left\{{Ñ}_{1},{Ñ}_{2},\cdots {Ñ}_{k}\right\}$. The *k* nodes in Ñ will construct *k* subtrees $T_{1},T_{2},\ldots ,T_{k}$.

Till now, this paper have partition the tree *T* into several subtrees $T_{1},T_{2},\ldots ,T_{k}$. The next operation is trying to balance the subtrees. Let $T^{max}$ and $T^{min}$ denote the trees with maximal and minimal numbers of sensor nodes, respectively. Let Θ(*T*) denote the number of nodes in tree *T*. The next step is to balance the tree size. The tree balancing can be achieved by moving some nodes of other tree to tree $T^{min}$ or moving some nodes from tree $T^{max}$ to other trees. To achieve this, the following defines neighboring trees which allow them to move node from one to another. Two trees $T_{i}$ and $T_{j}$ are said to be neighboring trees if there exist $\;s_{i}\in T_{i}$ and $\;s_{j}\in T_{j}$ such that $\;s_{i}$ and $\;s_{j}$ are neighbors. The following illustrates how the tree balancing can be achieved. Let *N*(*T*) denote the set of neighboring trees of tree *T*. Let sensor $s^{best}$ denote the sensor that will be moved to tree $T^{min}$. The sensor that satisfies Expression (14) will be selected to move to tree $T^{min}$.

(14) $$\;\;s^{best}=\;arg\underset{s_{i}\in T_{i},T_{i}\in N(T^{\min})}{\max}\frac{dis\left(s_{i},T_{i}\right)}{dis\left(s_{i},T^{min}\right)}\;$$

where dis(s,T) denotes the number of hops from sensor *s* to the root of tree *T*. That is, the sensor that has a large hop distance to the root of tree $T_{i}$ indicates that the energy consumed for packet forwarding of sensor is large. On the contrary, the sensor that has a small hop distance to the root of tree $T_{min}$ indicates that the energy consumed for packet forwarding of sensor is small. As a result, the sensor that meets the requirement of Expression (14) can reduce maximal energy consumption of tree $T_{i}$ and increase minimal energy consumption of tree $T^{min}$. The aforementioned operations must be repeated until the number of subtree nodes is uniform. That is *|*$T_{1}$*| = |*$T_{2}$*| =*⋯*= |*$T_{k}$*|.* The following presents the proposed *BTP* algorithm (Algorithm 1).

**Algorithm 1**. Balanced Tree Partition (*BTP*) Algorithm.  ***Input:***
$S=\left\{s_{1},s_{2},s_{3},\cdots s_{n}\right\}$, $\;T^{\prime }=\emptyset$;  ***Output:*** A set of balanced subtree $T^{\prime }=\left\{\;T_{1}^{'},\;T_{2}^{'}\cdots ,T_{k}^{'}\right\};$  1. Ñ={sink}  2. **While** ($\hat{S}\neq \emptyset \;$){  3.  Evaluate NP(i) according to Equation (10), for each $s_{i}\in S$;  4.   Evaluate D(i,T), according to Equation (11), for each $s_{i}\in S$;  5.  Evaluate $W_{i}\;\mathrm{according}\;\mathrm{to}\;\mathrm{Equation}\;\left(12\right)$;  6.   $\;s^{largest}=arg\underset{\;s_{i}\in \hat{S}}{\max}W_{i};$  7.   $Ñ=Ñ\cup \left\{s^{largest}\right\}\;;$  8.  $\hat{S}=\hat{S}-\left\{s^{largest}\right\}$;  9.  Reconstruct *k* subtrees $\;T=\left\{T_{1},T_{2},\cdots ,T_{k}\right\};\;$  10.  }  11. Let $nb_{i}$ = numbers of nodes in subtrees $T_{i}$, for 1 ≤ *I*
≤k;  12. $\;T_{avg}=\frac{\sum_{i=1}^{k}nb_{i}}{k}$;  13. **For** (*z* = 1; *z* <= *k*; *z* ++){  14.    **If** ($nb_{z}==T_{avg})${  15.     $T^{\prime }=T^{\prime }\cup \left\{T_{z}\right\};$  16.     *T*=*T* − $\left\{T_{z}\right\};$}  17.    **Else** {  18.    $T^{max}=max\left|T_{i}\right|,T_{i}\in \left\{T_{1},T_{2},\;\cdots ,T_{k}\right\}$;  19.    $T^{min}=min\left|T_{j}\right|,$
$T_{j}\in \left\{T_{1},T_{2},\;\cdots ,T_{k}\right\};$  20.     **If** ( $T_{i}$ and $T^{min}$ are neighboring trees)  21.      $\;s^{best}=arg\underset{s_{i}\in T_{i},T_{i}\in N\left(T^{\min}\right)}{\max}\frac{dis\left(s_{i},T_{i}\right)}{dis\left(s_{i},T^{min}\right)}\;\;;\;\;\;$  22.      Remove the edge linking from $s^{best}\;\mathrm{to}\;\mathrm{its}\;\mathrm{parent}$;  23.      Connecting point$\;s^{best}\;$to the nearest node in$\;T^{min}$;}}  24. $\;Return\;T^{\prime };$

**Figure 2:**
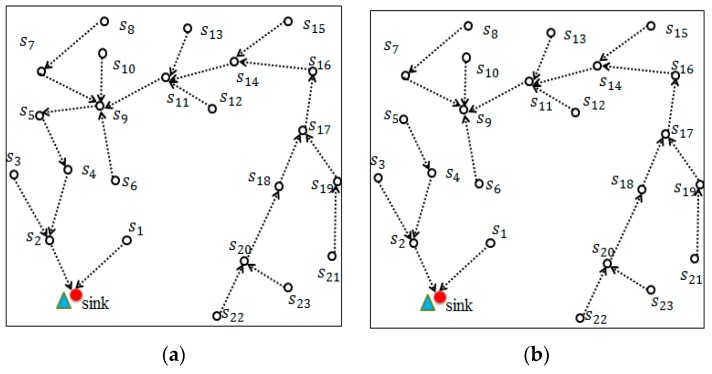

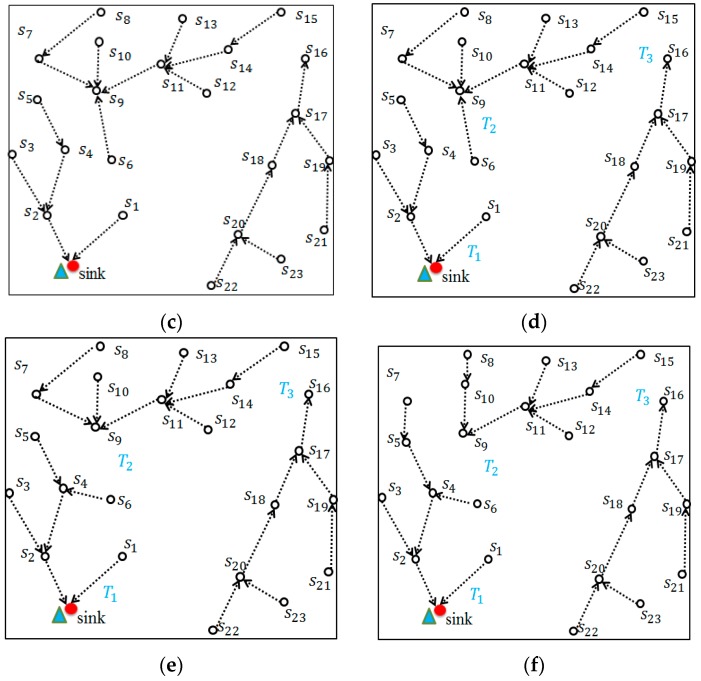
An example of network partition phase. (**a**) Given a minimal spanning tree; (**b**) Selecting sensor node $s_{9}$ as subroot; (**c**) Selecting sensor node $s_{16}$ as new root; (**d**) Three subtree $T_{1},T_{2}\;\mathrm{and}\;T_{3};\;$ (**e**) Selecting sensor node $s_{6}$ to move from $T_{2}$ to $T_{1}$; (**f**) Selecting sensor node $s_{7}$ to move from $T_{2}$ to $T_{1}$.

The following presents an example for operating the proposed *BTP* algorithm. The proposed *BTP* algorithm calculates the $W_{i}$ of each sensor node in tree and then selects $s_{9}$ which has the maximal value of cost index as the candidate of the new root. The *BTP* algorithm then adds $s_{9}$ into tour set Ñ and removes $s_{9}$ and edge ($s_{5},s_{9}$) from the minimal spanning tree given in Figure 2a. As a result, the original MST has been partitioned into two trees, as shown in Figure 2b. The repetitions of executing *BTP* algorithm will continuously select one sensor node with the maximal cost index value to play the role of new root until the number of nodes in Ñ reaches to *k*. In the second repetition, the proposed *BTP* algorithm will select $s_{16}$ to play the role of new root since $s_{16}$ has the maximal value of cost index. As shown in Figure 2c, tree *T* has been partitioned into three subtrees $T_{1},T_{2}\;\mathrm{and}\;T_{3}$.

Then *BTP* algorithm tried to balance the subtrees $T_{1},T_{2}\;\mathrm{and}\;T_{3}$.The numbers of nodes in subtrees $T_{1},T_{2}\;\mathrm{and}\;T_{3}$ are 6, 10, 8, respectively. The average number of nodes of the three subtrees is 8. To balance the three subtrees, $T_{2}$ should move two nodes to $T_{1}$. According to Equation (14), the proposed *BTP* algorithm will evaluate the distances from each sensor node in subtrees $T_{1}$ and $T_{2}$ to the roots of $T_{1}$ and $T_{2}$. As a result, sensor $s_{6}$ satisfies Expression (14) and will be selected to play the role of $s^{best}$. Hence an additional edge ($s_{6\;},s_{4})$ will be added in subtree $T_{1}$ and edge ($s_{6\;},s_{9\;}$) will be deleted from $T_{2}$. Repeat the abovementioned operations, until the sizes of three subtrees are identical. That is *|*$T_{1}$*| = |*$T_{2}$*| = |*$T_{3}$*|.* Figure 1f and Figure 2e depict the process of moving two nodes $s_{6}$ and$\;s_{7\;}$from $T_{2}$ to $T_{1}$.

After completing this phase, the mobile sink executes the CP Selection and Path Construction Phase which is described in the next subsection.

### 4.2. CP Selection and Path Construction Phase

This phase aims to select $k_{i}$ CPs from the sensors in *T_i_* and construct a path $\pi_{i}$ which visits the $k_{i}\;$ CPs for data collection.

Let $P_{i}$ denote the set of data collection points which has been selected to play the role of root in subtree $T_{i}$. Let $S_{i}$ denote the set of all sensors in subtree $T_{i}\;$and $S_{i}{}^{'}$ denote the set of sensors that are not selected to play the role of CPs. This phase firstly selects one best sensor node $s^{best}$ from set $S_{i}{}^{'}$ at a time. Then the selected $s^{best}$ will join the set $P_{i}$ and be removed from$\;S_{i}{}^{'}$. After that, the subtree will be restructured. Data collected by each node in set $P_{i}$ can be directly sent to the mobile sink $m_{i}$, since the mobile sink $m_{i}$ will visit each selected *CP* in set $P_{i}$. In this way, the energy consumption for those sensors that should forward the data of *CP* can be further saved.

Let $P_{i}=\left\{p_{i,1},p_{i,2},p_{i,3},\cdots ,p_{i,y}\right\}\;\;$denote the set of *y* CPs, which have been selected from in subtree $T_{i}$. Let $R_{i}$ denote the shortest Hamiltonian route the connects *y CP*s in $P_{i}$. Since the selection of each *CP* can construct a new subtree, it is obvious that subtree $T_{i}$ has been partitioned into *y* subtrees. Let $p_{i,1}\;$denote the root of tree $T_{i,j}$. Let $\;s_{x}$ be any sensor in tree $T_{i,j}$. Let $\;p^{closest}$ denote the CP which is closest to sensor node $\;s_{x}$. Equation (15) derives $\;p^{closest}$.

(15) $$\;\;p^{closest}=arg\underset{1\leq h\leq y}{min}\left(dist\left(p_{i,h},\;\;s_{x}\right)\right)\;$$

Let $b_{x}\;$denote the benefit index obtained by determining $\;s_{x}$ as the data collection point. The value of $b_{x}$, as derived in Equation (16), can be evaluated by the number of packets that are saved for transmission divided by the cost of distance from some $\;s_{i,j}\in P_{i}$ to$\;s_{i,i}$. 

(16) $$\;b_{x}=\frac{D\left(i,T\right)\;\times \;NP\left(i\right)}{dist\;p^{closest},\;s_{x}}\;$$

This phase will calculate $b_{x}\;\mathrm{for}$ each $s_{x}\in S_{i}{}^{'}$ and select the best node $s^{best}$ to play the role of *CP*, where $s^{best}$ satisfies the following condition. Equation (17) derives $\;s^{best}$.

(17) $$\;\;s^{best}=arg\underset{\;s_{x}\in T_{i,j}}{max}b_{x}\;$$

Then a new Hamiltonian route will be constructed by adding the new *CP* $s^{best}$ to the existing route $R_{i}$ for mobile sink $m_{i}$, where $R_{i}$ connects all (*y* + 1) *CP*s in set $P_{i}=P_{i}\cup \{s^{best}\}$ and checks whether or not the length $\left|\pi_{i}\right|$ is smaller than the length bound $L_{max}$. If it is the case, the proposed algorithm will add the collection point $s^{best}$ to set *P* and remove the $s^{best}\;$from set *V.* The edge that connects $s^{best}$ and its parent will be removed accordingly. Otherwise, the selection operation will be terminated. The set $\;P_{i}\;$ will be the output of this phase.

### 4.3. Speed Control Phase

This phase first constructs a global path which passes through each root of $T_{i}\;$for global mobile sink $m_{0}$ by applying the Hamiltonian algorithm. Let $T\;=\;\{T_{i}|1\;\leq i\;\leq k\}$ denote the set of *k* subtrees. The global mobile sink $m_{0}\;$ will leave from the static sink, move along the constructed path and collect all data stored in root $T_{i}$. Then the global mobile sink $m_{0}$ goes back to the static sink. Assume$\;\left\{\pi_{0},\pi_{1},\pi_{2},\ldots ,\pi_{k}\right\}$ denote the paths of the mobile sink $m_{0}$, $m_{1}$, *…*, $m_{k}$, respectively. Let $l_{i}$ denote the root location of $T_{i}$ which is also the rendezvous location of global mobile sink $m_{0}$ and mobile sink $m_{i}$.$\;\mathrm{Assume}\;\left\{{\hat{l}}_{0}\;,{\hat{l}}_{1},\;{\hat{l}}_{2},\ldots ,{\hat{l}}_{k}\right\}$ denote the order of rendezvous locations that the global mobile sink $m_{0}$ travel in a counter clockwise direction, where ${\hat{l}}_{i}$ be the rendezvous location of global mobile sink $m_{0}$ and local mobile sink $m_{i}$. Let$\;{\hat{t}}_{i}$ denote the rendezvous time point of every mobile sink $m_{i}$ and global mobile sink $m_{0}$. Assume $L_{i}$ denote the length from ${\hat{l}}_{i}$ to ${\hat{l}}_{i+1}$. The following presents how each $m_{i}$ adjusts its velocity such that it can meet $m_{0}$ at the rendezvous time point ${\hat{t}}_{i}$. Let *v* denote the velocity of the mobile sink $m_{0}$. The time required for mobile sink $m_{0}\;$ traverses path $L_{i}\;$ can be derived by applying Equation (18).

(18) $$\;t_{i}^{move}=\frac{L_{i}}{v}\;$$

Let $t_{i}^{collect}$ denote the time of the mobile sink $m_{0}$ collecting data in rendezvous point ${\hat{t}}_{i}$. The total time, denoted by $t_{i}^{arrive}$, required for global mobile sink $m_{0}\;$traversing from static sink to the rendezvous point ${\hat{l}}_{i}$ can be measured by Equation (19).

(19) $$\;\;\;\;t_{i}^{arrive}=t_{0}^{move}+t_{1}^{collect}+t_{1}^{move}+t_{2}^{collect}+\cdots t_{i-1}^{move}=\overset{i-1}{\underset{i=0}{\sum }}t_{i}^{move}+\overset{j-1}{\underset{i=1}{\sum }}t_{i}^{collect}\;$$

The total time, denoted by $t^{cycle}$, required for mobile sink $m_{0}$ touring a cycle can be measured by Equation (20).

(20) $$\;\;t^{cycle}=\;\frac{\sum_{i=0}^{k}L_{i}}{v}\;$$

The velocity of the mobile sink $m_{i}$ can be measured by Equation (21).

(21) $$\;\;v_{i}=\frac{\left|R_{j}\right|}{\;\;\;\;\;\;\;\;t_{j}^{arrive}}\;$$

where $\;\left|R_{i}\right|$ denote the path length of subtree *T_j_* passes through all collection point.

## 5. Performance Evaluations

This section aims to investigate the performance comparison of the proposed CDCA against the existing HiCoDG [20] and balanced regional partitioning algorithm BRPA [22] in terms of the rendezvous time, network lifetime, fairness index, the SD energy consumption and efficiency index. Four scenarios are considered in the experiments. Herein, the BRPA used a single mobile sink to collect the data from all CPs. To compared with the BRPA, the whole region is partitioned into several subregions and then each subregion applies the BRPA to select the CPs and allocate a mobile sink to collect data from the selected CPs.

Table 2 lists the parameters of the simulation environment. In the experiments, the sensor nodes are placed uniformly in an 800 m × 800 m rectangular region. The sensing range of each sensor is set by 20 m while the communication range is set by 30 m. The initial energy of each sensor is 100 J. Each sensor creates one data packet in each round which is a predefined time period for mobile sinks cooperatively passing through all rendezvous points and collecting data from all local mobile sinks. Assume that every mobile sink $m_{i}$ is aware of the time point that can meet the global mobile sink $m_{0}$.

**Figure 3:**
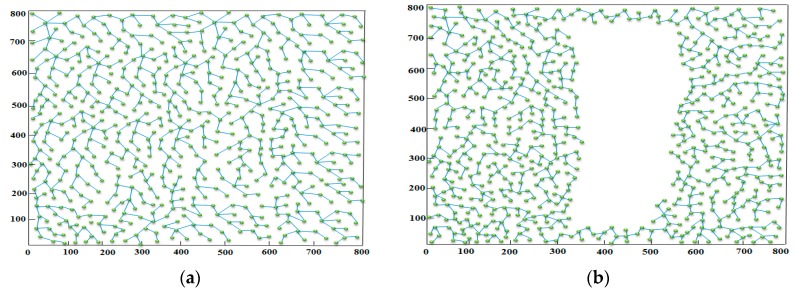

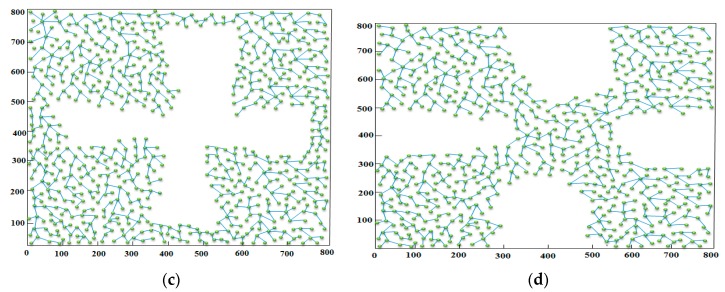
Four scenarios considered in the experiments. (**a**) BD-Scenario; (**b**) UD1-Scenario; (**c**) UD2-Scenario; (**d**) UD3-Scenario.

To further investigate the performance of the compared mechanisms in different distributions of the sensors, four scenarios are considered in the experiments, as shown in Figure 3. The first scenario, called balanced deployment scenario or BD-Scenario in short, adopts randomly deployment. All sensor nodes can communicate with each other. The other three scenarios are unbalanced deployment scenarios, called UD1-Scenario, UD2-Scen and UD3-Scenario. The big holes with circle-shape and X-shape are existed in the central region in UD1-Scenario and UD2-Scenario, respectively. In the UD3-Scenario, the sensors are connected to form an X-shape, which is partitioned by the holes. Figure 3b–d give instances of scenarios 2, 3 and 4, respectively, where the number of deployed sensors is 800.

**Figure 4:**
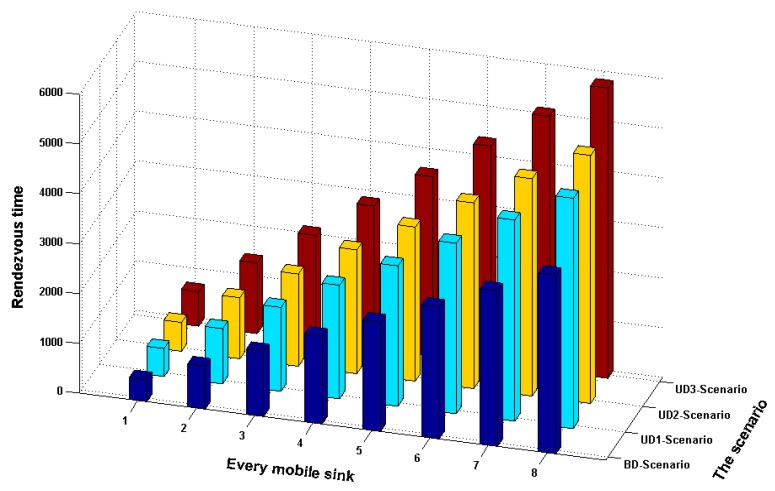
Comparison of the four scenarios in terms of rendezvous time.

Figure 4 shows the rendezvous time points between each of the eight local mobile sinks and the global mobile sink in four scenarios. In Figure 4, the four scenarios show the similar trend that the performance of BD Scenario is best and the rendezvous times are increased with the number of mobile sinks. In average, the BD Scenario saves 57% time cost, as compared with UD3-Scenario. Moreover, the UD2-Scenario and UD1-Scenario save 18% and 23% time costs, respectively, as compared with UD3-Scenario. The main reason is that BD Scenario has balanced deployment of sensors and contains no hole which might cause mobile sink moving from one side to another without any data collection operation, only for the purpose of travelling. As a result, the BD Scenario leads to a shorter path length. For each group, the proposed CDCA selects more CPs and creates shorter path length for each local mobile sink. Thus the average number of hops from each sensor to the CP and the average length between CPs is shorter. Consequently, the delay time of each local mobile sink is reduced. This also reduces the rendezvous time since the global mobile sink can rendezvous with each local mobile sink earlier.

**Figure 5:**
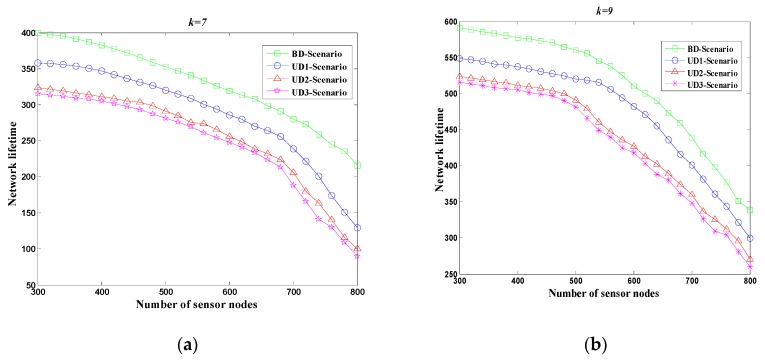
Network lifetime for four scenarios in different number of mobile sink. (**a**) *k* = 7; (**b**) *k* = 9.

Figure 5a,b investigate the network lifetimes of the four scenarios by varying the number of sensor nodes, ranging from 300 to 800 using seven and nine mobile sinks, respectively. By applying the proposed CDCA, the lifetime of WSN is increased with the number of mobile sinks. The BD-scenario has the longest network lifetime when the number of mobile sink is nine. This occurs because that more mobile sinks can visit more CPs and hence reduce average number of hops from each sensor to the corresponding CP. Since UD1-Scenario, UD2-Scenario and UD3-Scenario have holes which block data transmissions. As a result, the BD-scenario has the best performance in terms of network lifetime.

**Figure 6:**
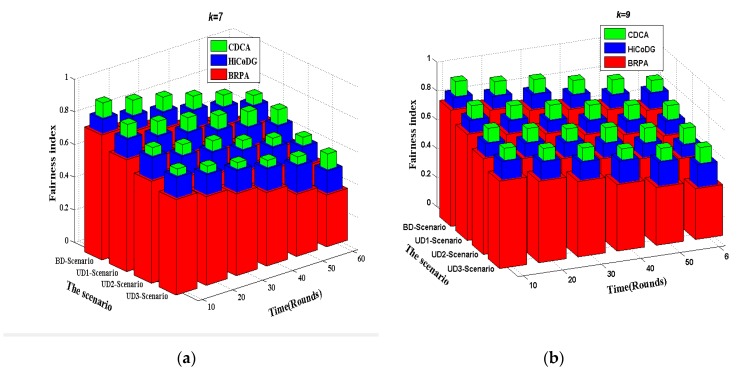
Fairness index. (**a**) *k* = 7; (**b**) *k* = 9.

Figure 6 compares the three algorithms in four scenarios in terms of fairness index. Let $E_{i}\;$denote energy consumption of sensor node $s_{i}$ in each round. The *Fairness Index* is defined by Expression (22).

(22) $$\;\;\tau_{fairness}=\frac{{\left(\sum_{i=1}^{n}E_{i}\right)}^{2}}{n\sum_{i=1}^{n}E_{i}^{2}}\;$$

As shown in Figure 6, the fairness indexes of three approaches, including CDCA, HiCoDG and BRPA, are compared. The BRPA approach partitions the monitoring region into several equal sub-regions and assigns each mobile sink to collect all sensors in each subregion. Since different sub-regions have different number of sensors, it has smaller fairness index value. In HiCoDG, the balance of the number of nodes in clusters is not considered. Furthermore, the distance from the current cluster-head to the next CP is not considered. Therefore, more overheads are needed for travelling from one CP to another one. As a result, fewer CPs can be joined, leading to a larger hop distance from each sensor to the corresponding CP. The proposed CDCA determines more CPs and distributes the workloads of data forwarding to them. Hence the fairness index of CDCA keeps to a constant value which is closed to 1 in all cases. In general, the proposed CDCA outperforms HiCoDG and BRPA schemes in terms of fairness index in all cases.

The standard deviation (SD) of the energy consumption of each sensor node was measured using Equation (23).

(23) $$\;\mathrm{SD}=\sqrt{\frac{\sum_{1}^{n}{\left(E_{x}\;-\;E_{avg}\right)}^{2}}{n}}\;$$

where $E_{x}$ denotes energy consumption of sensor node $s_{i}$ and $E_{avg}$ denote average energy consumption of sensor node. A small SD value indicates that the network lifetime is long.

**Figure 7:**
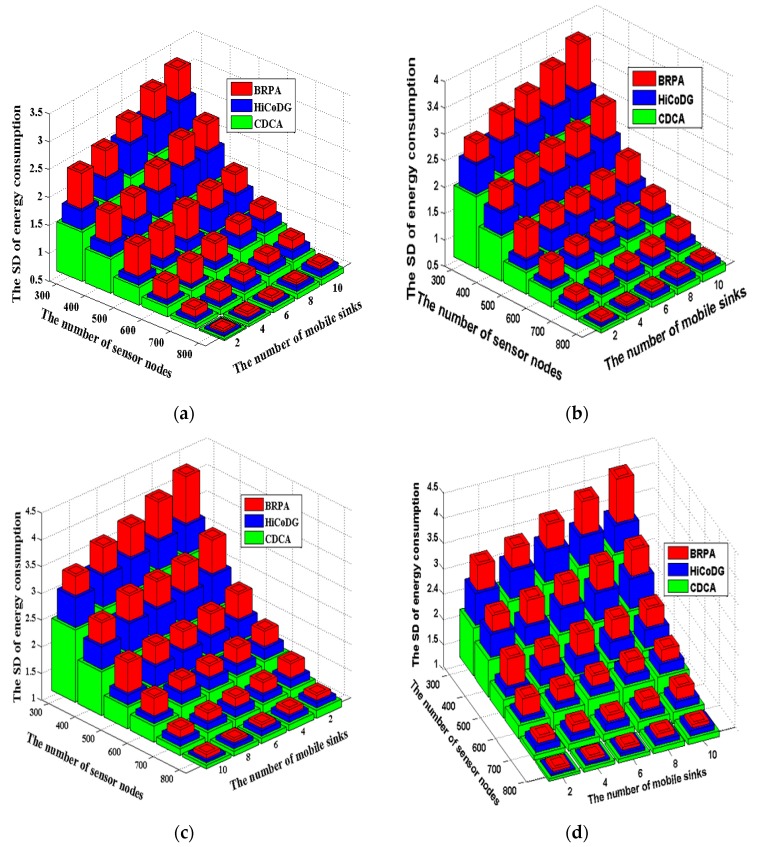
**Figure7.** The comparison of SD energy consumption of three algorithms. (**a**) BD-Scenario; (**b**) UD1-Scenario; (**c**) UD2-Scenario; (**d**) UD3-Scenario.

Figure 7 further investigates the effects of the numbers of sensor nodes and mobile sinks on the SD value in four scenarios by applying three mechanisms. The number of sensor nodes was varied ranging from 300 to 800, and the number of mobile sinks was set ranging from 2 to 10. In general, the SD value is decreased with the number of sensor nodes. The BRPA yielded the largest SD value among the three compared mechanisms because the node balance in each subtree was not considered. For the HiCoDG scheme, multiple mobile sinks cooperate with each other to collect and relay data, resulting in a lower SD value, as compared with the existing BRPA. However, the SD value of the HiCoDG scheme was higher than that of the proposed CDCA mechanism. This occurs because it did not consider the node balance factor in each cluster. Moreover, the distance from the current cluster-head to the next cluster-head is not considered when constructing the path. The proposed CDCA mechanism partitions the sensor nodes into several disjoint subsets, and further selects as more as possible CPs in $T_{i}$. Then CDCA constructs a path $\pi_{i}$ passing through all CPs and collects data from higher burden CPs in each subset. After that, the CDCA further applies the speed control mechanism to maintain a stable rendezvous time point for global mobile sinks $m_{0}$ and each mobile sink $m_{i}$. As a result, the proposed CDCA mechanism yielded the lowest SD value, as shown in Figure 6.

**Figure 8:**
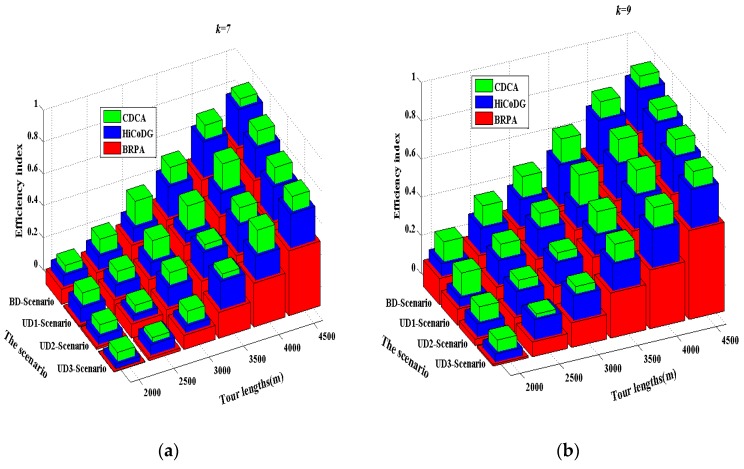
The comparison of efficiency index of three algorithms in four scenarios. (**a**) *k* = 7; (**b**) *k* = 9.

Let $\zeta_{efficiency}$ represent the efficiency index equal to the average length cost multiplied by the resource unused rate. The path length cost is measured by the total length $l_{at}$ divided by the number of CPs (*τ*). The maximal length of path constraint is considered as the resource and the unused resource is measured by $1\;-\;\left(l_{at}/l_{max}\right)$. Equation (24) indicates that the efficiency index depends on the path length cost for each CP and the resource unused rate.

(24) $$\;\;\zeta_{efficiency}=1\;-\;\left[\left(\frac{l_{at}}{\tau }\right)\;\times \;\left(1\;-\;\frac{l_{at}}{l_{max}}\right)\;\right]\;$$

where $l_{at}$ denotes the actual length of mobile sink $m_{i}$ travelling path in each subtree in each round and *τ* denotes the number of CP in subtree. Figure 8 depicts the impact of path length on the efficiency index. Four scenarios were considered in the simulation. In general, the efficiency indices of the BRPA, HiCoDG and CDCA mechanisms increase with the number of mobile sinks, as shown in Figure 8a,b The proposed mechanism CDCA outperforms the other two approaches HiCoDA and BRPA. This occurred because that HiCoDA and BRPA mechanisms do not consider the constraints of number of mobile sinks and path length. As shown in Figure 8, the ERPA yielded the smallest efficiency index value among the three compared mechanisms because the node balance in different subtrees was not considered. In addition, the efficiency index value of HiCoDG scheme was smaller than that of the proposed CDCA mechanism. This occurs because that the HiCoDG scheme also did not consider the node balance issue in different clusters and the distance between current cluster-head and the next cluster-head is not taken into consideration. The proposed CDCA balances the node number in different subtrees and finds as more as possible CPs under the constraint of total path length in each subtree. Therefore, the proposed CDCA achieves higher resource utilization and smaller path cost, as compared with the other two mechanisms. Consequently, the proposed CDCA mechanism yielded the largest efficiency index value.

## 6. Conclusions

This paper considers the data collection issue in a given mobile wireless sensor network where multiple mobile sinks aim to collect data from a given set of static sensors. The proposed CDCA mechanism aims to prolong the network lifetime by visiting as more CPs as possible such that the forwarding cost of each static sensor is reduced. The proposed CDCA mechanism primarily consists of three phases: Network Partition Phase, CP Selection and Path Construction Phase, and Speed Control Phase. In the Network Partition Phase, the CDCA partitions the sensor nodes into *k* disjoint subsets. In the Collection Point Selection Phase and Path Construction Phase, CDCA further selects $k_{i}$ CPs from sensors in $T_{i}$ and constructed a path $\pi_{i}$ which passes through the $k_{i}$ CPs and collects data from higher burden CPs. In the Speed Control Phase, the algorithm coordinates to control the speed of mobile sinks such that the global sink and each local mobile sink can be rendezvoused. The performance results show that the proposed CDCA outperforms other mechanisms in terms of rendezvous time, network lifetime, fairness index, standard deviation (SD) of energy consumption, as well as efficiency index. The proposed CDCA reduces the energy consumption of tested WSNs by 18% and 37% in comparison to HiCoDG and BRPA, respectively.

Further studies for exploring energy recharging are planned. The large WSN is partitioned into smaller areas where each area is assigned a mobile sink. The proposed CDCA can be extended such that it can establish a recharging path for each area while considering the coverage issue. Moving along the path, a mobile recharger can recharge the sensors for maintaining maximal coverage for a given WSN.

## Figures and Tables

**Table 1:** Comparison between the proposed CDCA and existing mechanisms.

Studies	Number of Mobile Sink	Path Construction	Data Collection Latency	Mobile Sinks Cooperation
[14]	single	O	long	×
[15]	single	O	long	×
[16]	multiple	O	short	×
[17]	multiple	×	long	O
[18]	multiple	×	short	×
[19]	multiple	×	short	×
[20]	multiple	O	short	O
The proposed CDCA	multiple	O	short	O

**Table 2:** Simulation setting.

Parameter	Value
Node deployment	Uniform random distribution
Given region	800 m × 800 m
The number of sensor node	600–800
Mobile sink speed	3 m/s
Sensor node transmission range	30 m
Consumed energy in transmitter circuit	0.18 J
Consumed energy at the receiver circuit	0.1 J
